# Lingguizhugan oral solution alleviates MASLD by regulating bile acids metabolism and the gut microbiota through activating FXR/TGR5 signaling pathways

**DOI:** 10.3389/fphar.2024.1426049

**Published:** 2024-08-15

**Authors:** Jiahua Wang, Juan Zang, Yang Yu, Yang Liu, Huimin Cao, Ruibo Guo, Lu Zhang, Mo Liu, Zixu Zhang, Xuetao Li, Liang Kong

**Affiliations:** ^1^ College of Pharmacy, Liaoning University of Traditional Chinese Medicine, Dalian, China; ^2^ Key Laboratory of Ministry of Education for Traditional Chinese Medicine Viscera-State Theory and Applications, Liaoning University of Traditional Chinese Medicine, Shenyang, China

**Keywords:** Lingguizhugan oral solution, metabolic dysfunction-associated steatotic liver disease, gut microbiota, bile acid metabolism, FXR/TGR5

## Abstract

**Background:**

The preservation of the Lingguizhugan (LGZG) decoction and patient compliance issue often limit the treatment of metabolic dysfunction-associated steatotic liver disease (MASLD). Hence, herein, an LGZG oral solution was developed for alleviating MASLD. Additionally, the potential mechanisms underlying LGZG-mediated MASLD mitigation were explored.

**Methods:**

A MASLD mouse model was constructed using oleic and palmitic acid-induced LO2 cells and a high-fat diet. The apoptosis, lipid deposition, and mouse liver function were analyzed to assess the therapeutic effects of the LGZG oral solution on MASLD. Serum untargeted metabolomics, gut microbiota, bile acid (BA) metabolism, immunohistochemistry, and Western blotting analyses were performed to investigate the potential mechanism of action of LGZG oral solution on MASLD.

**Results:**

The LGZG oral solution ameliorated lipid deposition, oxidative stress, inflammation, and pathological damage. Serum untargeted metabolomics results revealed the LGZG-mediated regulation of the primary BA biosynthetic pathway. The 16S ribosomal RNA sequencing of the fecal microbiota showed that LGZG oral solution increased the relative abundance of the BA metabolism-associated *Bacteroides*, *Akkermansia*, and decreased that of *Lactobacillus*. Additionally, the BA metabolism analysis results revealed a decrease in the total taurine-α/β-muricholic acid levels, whereas those of deoxycholic acid were increased, which activated specific receptors in the liver and ileum, including farnesoid X receptor (FXR) and takeda G protein-coupled receptor 5 (TGR5). Activation of FXR resulted in an increase in short heterodimer partner and subsequent inhibition of cholesterol 7α-hydroxylase and sterol regulatory element-binding protein-1c expression, and activation of FXR also results in the upregulation of fibroblast growth factor 15/19 expression, and consequently inhibition of cholesterol 7α-hydroxylase, which correlated with hepatic BA synthesis and lipogenesis, ultimately attenuating lipid deposition and bile acid stasis, thereby improving MASLD.

**Conclusion:**

Altogether, the findings of this study suggest that modulating microbiota–BA–FXR/TGR5 signaling pathway may be a potential mechanism of action of LGZG oral solution for the treatment of MASLD.

## 1 Introduction

Metabolic dysfunction-associated steatotic liver disease (MASLD), previously known as non-alcoholic fatty liver disease (Castro-Narro and Rinella, 2024; Hsu and Loomba, 2024; Lee et al., 2024), is the most common metabolic syndrome and chronic liver disease worldwide characterized by hepatocyte steatosis and fat accumulation (Huang et al., 2020). Reportedly, MASLD is a prominent cause of liver-related morbidity and mortality, affecting approximately 25% of adults worldwide (Raza et al., 2021). Patients with MASLD are primarily treated by mitigating or halting the disease progression through lifestyle adjustments and dietary optimization. Additionally, caloric restriction for weight reduction is considered an effective therapeutic strategy for MASLD (Liu et al., 2019). Regarding the pharmacologic treatment of MASLD, current international guidelines only consider a few pharmacologic approaches including vitamin E and pioglitazone. Reportedly, pharmacologic agents such as glucagon-like peptide one agonists, farnesoid X receptor (FXR), and peroxisome proliferator-activated receptor ligands have exhibited beneficial effects on MASLD in clinical trials, although they are associated with certain limitations (Paternostro and Trauner, 2022). Therefore, studies on the multi-pathway, multi-compound, multi-target treatment model of traditional Chinese medicine (TCM) are urgently warranted.

TCM presents several advantages in treating chronic liver diseases, including precise therapeutic effects and minimal adverse reactions. Lingguizhugan (LGZG) decoction is a classic formula documented in the “Synopsis of Golden Chamber” and consists of the following four TCMs: Fuling, Wolfiporia cocos (F.A. Wolf) Ryvarden and Gilb. [Polyporaceae; Poria]; Guizhi, Cinnamomum cassia (L.). J. Presl [Lauraceae; Cinnamomi ramulus]; Baizhu, Atractylodes macrocephala Koidz. [Asteraceae; Atractylodis macrocephalae rhizoma] and Gancao, Glycyrrhiza uralensis Fisch. ex DC. [Fabaceae; Glycyrrhizae radix et rhizoma]. LGZG decoction can warm Yang, transform drink, strengthen the spleen, and dispel dampness. The effects of LGZG decoction in treating various liver diseases such as MASLD (Cao et al., 2022), heart diseases such as heart failure (Li et al., 2019), and spleen disorders such as diarrhea (Xu et al., 2020) have been reported in many clinical trials. Regarding MASLD treatment, this decoction considerably improved the liver function and blood lipid levels, along with lipid metabolism regulation (Zhu et al., 2017), oxidative stress (Yang et al., 2017), and inflammation to prevent disease progression (Cao et al., 2022). Nevertheless, despite these positive outcomes, the exact therapeutic mechanism of LGZG decoction remains unelucidated.

MASLD pathogenesis is multifaceted and not fully understood. Recently, bile acids (BAs), gut microbiota, nuclear receptors (NRs) including FXRs and liver X receptors, lipid metabolism, and fatty acid metabolism have been reported to play a driving role in preventing and treating MASLD (Yan et al., 2018; Chen and Vitetta, 2020; Bing and Li, 2022). Among them, abnormal BA metabolism is considered an important contributor to MASLD development. FXR is a metabolic NR, also known as BA receptor, and maintains BA homeostasis (Keitel et al., 2019a). Takeda G protein-coupled receptor 5 (TGR5) is another important BA receptor and is activated by BAs to regulate their metabolism (Castellanos-Jankiewicz et al., 2021). FXR is highly expressed in hepatocytes and intestinal epithelial cells and is involved in BA synthesis, excretion, and reabsorption (Molinaro and Marschall, 2022). Notably, FXR functions differently in the liver and intestine to regulate BA homeostasis. In the liver, FXR activation modulates short heterodimer partner (SHP) to suppress cholesterol 7α-hydroxylase (CYP7A1) expression, thereby inhibiting BA synthesis (Chiang and Ferrell, 2020). In the intestine, FXR activation induces fibroblast growth factor 15/19 (FGF15/19) to maintain BA homeostasis (Katafuchi and Makishima, 2022). Reportedly, FXR receptors can regulate BA metabolism (Clifford et al., 2021), intestinal flora homeostasis (Zhai et al., 2022), and other signaling pathways for treating MASLD, making them a promising therapeutic target for fatty liver and related conditions.

The gut microbiota exhibits a highly intricate composition, comprising approximately 500–1,000 species, with nearly 10^14^ bacteria, which is over ten times the total number of cells in the human body (Ma et al., 2019). This microbial population plays a crucial role in regulating metabolic processes and disease, and dysbiosis in the gut microbiota can alter the immune status of the body, contributing to the development of various liver diseases, including MASLD (Fang et al., 2022). An essential reaction in BA metabolism is the bile salt hydrolase (BSH)-catalyzed uncoupling of BAs by the gut microbiota. Reportedly, bacterial genera such as *Lactobacillus*, *Bacteroides*, *Bifidobacterium*, *Clostridium*, *Listeria*, and *Enterococcus* can exhibit functional BSH activity (Cai et al., 2022). The gut microbiota regulates the BA-pool homeostasis by metabolizing primary BAs into secondary BAs (Chen and Vitetta, 2020). The BA–gut microbiota interaction provides a rationale for exploring the potential of gut microbiota-targeted therapy of MASLD.

This study aimed to investigate the effects of the LGZG oral solution on oleic acid (OA)- and palmitic acid (PA)-induced LO2 cells and an MASLD mouse model. Serum untargeted metabolomics techniques were used to assess the differential metabolites. Furthermore, the changes of gut microbiota and BA profile were monitored by 16S ribosomal RNA (rRNA) sequencing and BA-targeted metabolomics to further explore the effect of LGZG oral solution on BA metabolism and lipid accumulation through FXR/TGR5 signaling pathway. Additionally, the potential mechanisms of the protective effects of LGZG oral solution in MASLD mice were elucidated.

## 2 Material and methods

### 2.1 Preparation of LGZG oral solution

Fuling, Guizhi, Baizhu and Gancao were supplied by Liaoning University of Traditional Chinese Medicine, School of Pharmacy, and verified by Professor Zhang Hui, Department of Chinese Medicine Identification, School of Pharmacy, Liaoning University of Traditional Chinese Medicine. Firstly, the volatile oil, the main compounds of the two botanical drugs, Guizhi and Baizhu, was extracted by water vapor distillation, with the amount of water added being 8 times that of the botanical drugs, and the extraction time was 6 h, and the volatile oil was collected. Then the water decoction method was used to extract the botanical drugs (4:3:3:2) twice at a ratio of 1:8 botanical drugs to water for 1.5 h each time. Then, the extract was clarified by precipitation with 70% ethanol for 18 h, The ethanol was volatilized so that its PH was between 4 and 6, and then the flavoring agent (mannitol), and preservative (potassium sorbate) were added. After that, we mixed the co-solvent 1.5% Tween 80 with the volatile oil, so that the volatile oil was fully emulsified, and then the emulsion was added to the previous medicinal solution, stirred to dissolve, and finally made into LGZG oral solution.

HPLC was performed to identify the major compounds in LGZG oral solution. Chromatographic separation was performed using an Shim-park GIST C18 column (4.6 mm × 250 mm, 5 μm, Shimadzu). The mobile phase was acetonitrile (A)-0.1% phosphoric acid (B). The gradient program was set as follows: 0–10 min, 5%–20% A; 10–40 min, 20%–50% A; 40–50 min, 50%–60% A; 50–75 min, 60%–70% A; 75–100 min, 70%–90% A; 100–110 min, 90%–20% A. Flow rate: 1 mL/min, wavelength: 254 nm, column temperature: 30°C.

### 2.2 Animals and materials

Male mice, weighing 14–20g, were purchased from Liaoning Changsheng Biotechnology Co., LTD. Mice were housed at the Laboratory Animal Center of Liaoning University of Traditional Chinese Medicine at 22°C–24°C and 55%–60% humidity. All experimental procedures were carried out in accordance with the institutional standards of animal humanistic care. The procedures followed in this study were approved by the Animal Research Ethics Committee of Liaoning University of Traditional Chinese Medicine, with which the informed consent for clinical research was signed (NO. 210000420230204). The research was conducted in accordance with internationally accepted principles for the use and care of laboratory animals. The mice were randomly divided into five groups (n = 10): control group, model group, LGZG-L treatment group, LGZG-M treatment group and LGZG-H treatment group. Mice were treated with a 4-week intervention starting at week 14, the LGZG-L (2.5 g/kg/d), LGZG-M (5.0 g/kg/d) and LGZG-H (10.0 g/kg/d) were administrated by gavage, respectively. The mice in the control group and the model group were given the same amount of normal saline by gavage. Body weight was monitored weekly, and mice were executed after 4 weeks of intervention. Blood samples were collected by enucleation of eyeballs and immediately centrifuged at 4°C for 10 min to obtain serum. Liver and ileum tissues as well as mouse cecum contents were also collected. Livers were weighed to derive a liver index and then stored at −80°C for further use.

Oleic acid (OA) and Palmitic acid (PA) was obtained from Shanghai Aladdin Biochemical Technology Co.,Ltd (Shanghai, China). Oil red O staining solution was bought from Solarbio Science and Technology Co., Ltd (Beijing, China). Hematoxylin and eosin (HE) staining kit, Annexin V/FITC apoptosis detection kit were ordered from the Meilun Biotechnology Co., Ltd (Dalian, China). Fetal bovine serum (FBS) and Penicillin-streptomycin (P/S) were purchased from Gibco BRL (Grand Island, NY, United States). TG and GSH-PX kits were obtained from Nanjing Jiancheng Bioengineering Institute (Nanjing, China). γ-GT kits were obtained from Nanjing Jiancheng Bioengineering Institute (Nanjing, China) and Solarbio Science and Technology Co., Ltd (Beijing, China). TC, HDL-C, LDL-L, AST, ALT, MDA and SOD kits were obtained from Pulilai gene Technology Co., Ltd (Beijing, China). BODIPY 493/503 from Fushen Biotechnology Co., Ltd (Shanghai, China). IL-6, IL-1β, and TNF-α kits from Solarbio Science and Technology Co., Ltd (Beijing, China). FXR and FGFR4 were obtained from proteintech Co., Ltd (Wuhan, China). TGR5 and FGF15 were obtained from abcam Co., Ltd (Shanghai, China). CYP7A1, CYP8B1, SREBP-1C and SHP were obtained from ThermoFisher Co., Ltd (Shanghai, China).

### 2.3 Cell culture, viability assay, and LO2 cell treatment

LO2 cells were cultured in DMEM supplemented with 10% fetal bovine serum, 100 units/mL penicillin, and 100 μg/mL streptomycin and maintained at 37°C with 5% carbon dioxide and 95% air. Cell viability was determined by SRB assay. Before administration, all liquid medicines were filtered through a 0.22 μm sterile membrane to remove bacteria. In order to induce lipid accumulation, OA and PA were added into the culture medium, and the cells were divided into control group, model group (OA:PA = 2:1), LGZG-L treatment group, LGZG-M treatment group, and LGZG-H treatment group.

### 2.4 Oil red O staining

Cells were stained with oil red O for determination of lipid content. Cells were washed twice with PBS, fixed with 4% paraformaldehyde for 30 min, incubated with oil red O working solution at room temperature for 30 min, and destained with 60% isopropanol. After washing three times with PBS, the cells were counterstained with hematoxylin for 30 s. Cells were photographed under a light microscope (Nikon, Tokyo, Japan) and to quantify the content of oil red O, 100 μL of isopropanol was added to each well, and its absorbance at 520 nm was measured by a microplate reader (Synergy H1, BioTek, United States).

### 2.5 BODIPY 493/503 staining method

The accumulation of intracellular lipid droplets was detected by BODIPY 493/503 staining. The cells were fixed with 4% paraformaldehyde for 30 min, followed by washing once with PBS, adding 150 μL and staining with 1 μM BODIPY 493/503 at 37°C for 30 min, and the cells were observed under a fluorescence microscope.

### 2.6 The apoptosis of LO2 cells was detected by flow cytometry

Briefly, cells were harvested and incubated with 100 μL Annexin V FITC conjugate and 5 μL PI Journal Pre-proof eight solution for 20 min in the dark. Cells were then resuspended in 200 μL PBS and detected by flow cytometry (BD Biosciences, Franklin Lakes, NJ).

### 2.7 Biochemical analysis

LO2 cells were treated as before, and each group of cells was collected. The levels of TG, GSH-PX, SOD, CAT, MDA, ALT, AST and γ-GT were measured by corresponding kits. And the serum levels of ALT, AST, TC, TG, HDL-C and LDL-C were detected by using the corresponding kits and according to the instructions.

### 2.8 Liver histological examination

The degree of fatty change in liver tissue after HE staining were observed by light microscopy. NAS score was used to assess the severity of hepatic inflammation, necrosis, and fibrosis. MASLD was categorized into three stages, NAFL (scores <3), borderline NASH (scores 3–4), and NASH (scores >5) (Yang et al., 2023). Oil red O staining was used to detect fat deposition in liver tissue.

### 2.9 Hepatic proinflammatory factors and antioxidant indexes were determined

Liver tissue homogenate was collected to detect the levels of proinflammatory cytokines IL-6, IL-1β, and TNF-α in the liver by ELISA. SOD and GSH-Px activities and MDA level in liver tissue homogenate were detected according to the instructions of SOD, MDA and GSH-Px kit.

### 2.10 Serum untargeted metabolomics study

The 50 mg sample was slowly thawed on ice and placed in a 2 mL centrifuge tube. Subsequently, 800 μL of 80% methanol was added, followed by grinding at 65 Hz for 90 s and mixing with vortex shaking. The sample was then subjected to ultrasound at 4°C for 30 min, and allowed to stand for 1 h at −20°C. After centrifugation at 12,000 rpm/min for 15 min at 4°C, 200 μL of the supernatant was extracted. To this, 5 μL of 0.14 mg/mL dichlorophenylalanine was added as the internal standard, mixed, and transferred into sample vials for LC-MS/MS analysis.

The chromatographic platform utilized in this study was the Ultimate 3000 L C combined with Q Exactive MS (Thermo). The chromatographic column employed was the ACQUITY UPLC HSS T3 column (2.1 mm × 100 mm, 1.8 μm). The separation conditions included a column temperature of 40°C, a flow rate of 0.3 mL/min, and mobile phase A consisting of 0.05% formic acid-water, while mobile phase B was acetonitrile. The injection volume was 6 μL, and the autosampler temperature was maintained at 4°C. Mass spectrometry was conducted in positive ion mode with a heater temperature of 300°C. The sheath gas flow rate was set at 45 arb, auxiliary air flow rate at 15 arb, and exhaust flow rate at one arb. The electrospray voltage was 3.0 KV, capillary temperature at 350°C, and S-LensRFLevel at 30%.

### 2.11 16sRNA sequencing of the fecal microbiota

Total fecal DNA samples were extracted for 16S rRNA sequencing. PCR amplification was then performed. High-throughput sequencing was performed on Illumina Novoseq6000 PE250. Using QIIME software, the UCLUST Sequence Comparison Tool (Edgar, 2010) was invoked to analyze the obtained sequences by 97% similarity for operational taxonomic units (OTU) clustering and species taxonomy.

### 2.12 Analysis of fecal bile acids

Samples were accurately weighed and recorded before being loaded into 1.5 mL EP tubes along with 10 μL of mixed internal standard and 390 μL of methanol containing 1 mM BHT. Two small steel balls were added, followed by swirling for 30 s, resting at −20°C for 2 min, and then grinding in an ice bath using a grinder at 60 Hz for 1 min. Ultrasonic extraction was performed for 10 min, followed by centrifugation at 4°C and 12,000 r for another 10 min. The supernatant was then diluted 10-fold, with 100–150 μL being bottled for further analysis. The experiment utilized the UPLC-ESI-MS/MS analysis method for qualitative and quantitative detection of target metabolites.

Chromatographic conditions: injection volume: 5 μL; flow rate: 0.45 mL/min: mobile phases A (0.1% formic acid-water solution), B (methanol: ethanol: isopropanol = 1:1:1, containing 0.1% formic acid). Gradient Elution Procedures: 0 min A/B (80:20, V/V), 0.5 min A/B (80:20, V/V), 1.5 min A/B (62:38, V/V), 12 min A/B (50:50, V/V), 17.5 min A/B (5:95, V/V), 19 min A/B (5:95, V/V), 19.01 min A/B (80:20, V/V), 20 min A/B (80:20, V/V). Mass spectrometry method conditions: curtain gas: 35 (psi), collision-activated dissociation (CAD) parameters: medium, negative ion spray zero voltage: 4500 V, positive ion spray voltage: 5500 V, ion source temperature: 450°C, column temperature: 45°C, spray gas (Gas1): 55 (psi), auxiliary heating gas (Gas2): 55 (psi).

### 2.13 Western blot

An appropriate amount of liver tissue stored at −80°C was used to extract protein with RIPA lysate. After centrifugation at 4°C for 20 min, the protein concentration was measured and denatured by boiling. The denatured protein samples were separated by electrophoresis, transferred to the membrane, and closed. Dilutions of primary antibodies CYP7A1, CYP8B1, FXR, TGR5, SHP, SREBP-1C and FGFR4 (1:1,000, v:v) were added and incubated at 4°C overnight. The corresponding secondary antibody dilution (1:1,000, v:v) was added and incubated for 1 h at room temperature. The ECL mixture was drip-added onto the membrane, exposed by chemiluminescence imaging system, photographed by gel imager after development, and analyzed by ImageJ software.

### 2.14 Immunofluorescence and immunohistochemistry

Liver/ileum paraffin sections were routinely dehydrated, placed in 0.01 mol/L sodium citrate buffer, microwaved to boiling for 5 min, and repeated 3 times; the sections were incubated in 3% H_2_O_2_ solution for 5 min at room temperature; permeabilized in 1% Triton X-100 solution for 30 min; and occluded in 5% BSA solution for 1 h. FXR, TGR5 and FXR, TGR5, FGF15/19 primary antibody dilution (1:300) was respectively added to liver and ileum tissues in drops of 50 μL and incubated at 4°C overnight; the next day, Cy3/FITC labeled secondary antibody dilution (1:300) was added in drops, and the nuclei of the cells were stained with DAPI for 10 min, and the positive expression was observed under fluorescence microscope. HRP-labeled secondary antibody dilution (1:300) was added dropwise for immunohistochemical staining, incubated at room temperature and protected from light for 1 h, DAB color development, hematoxylin re-staining, dehydrated with different gradient ethanol solutions, sealed with xylene clear neutral gum, and observed the positive expression under the microscope.

### 2.15 Statistical analysis

Statistical analysis was performed using GraphPad Prism 9.0 software, and data were expressed as mean ± standard deviation (mean ± SD). Comparisons between groups were analyzed by one-way ANOVA or *t*-test, with *p* < 0.05 indicating a statistically significant difference.

## 3 Results

### 3.1 Identification of chemical compounds in the LGZG oral solution

**FIGURE 1:**
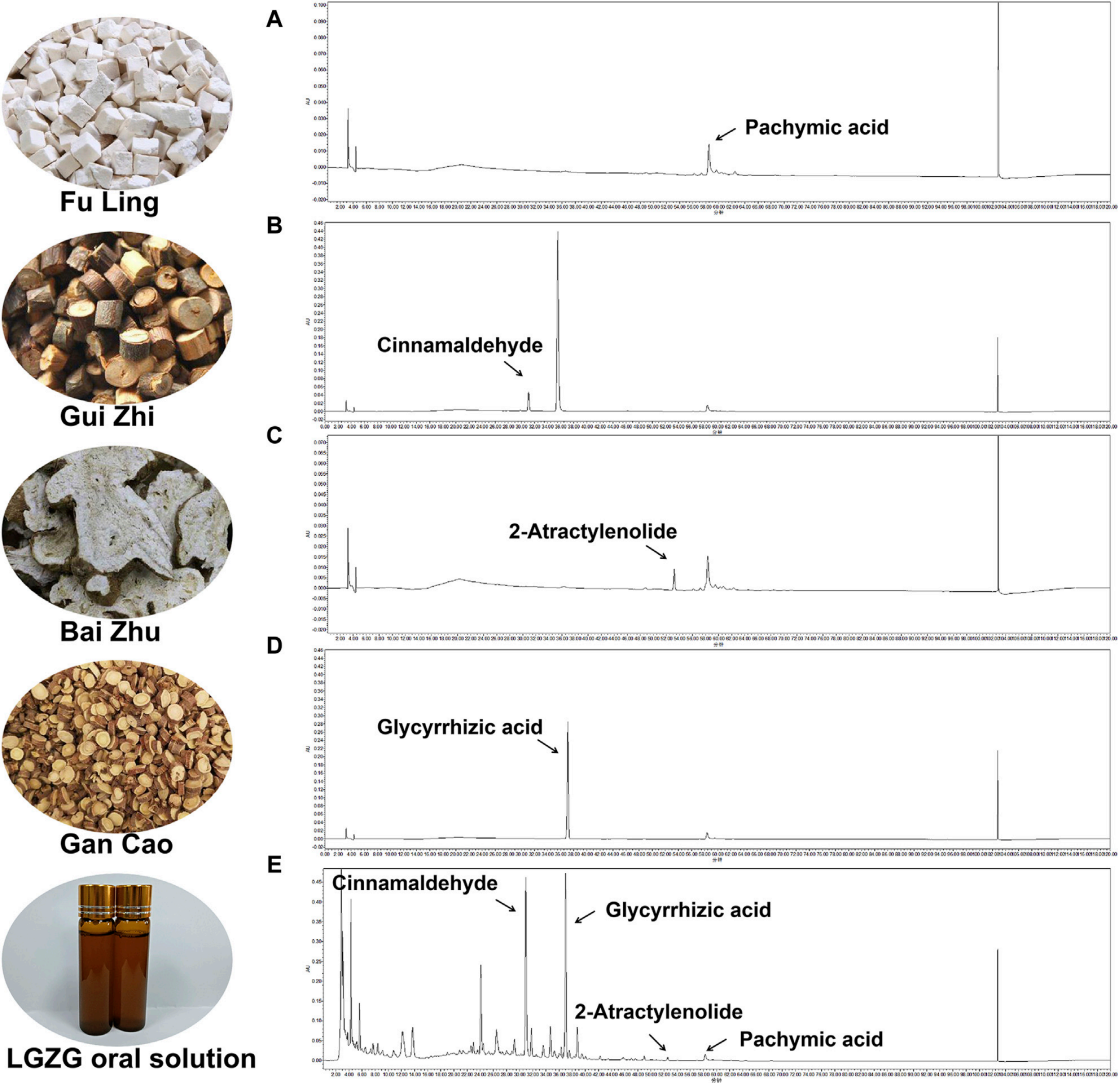
Identifcation of chemical compounds in LGZG oral solution by HPLC. (**A–D)** HPLC chromatograms of Cinnamaldehyde, Glycyrrhizic acid, 2-Atractylenolide and Pachymic acid at 254 nm. **(E)** HPLC chromatograms of the LGZG oral solution at 254 nm.

The chemical compounds of LGZG oral solution was characterized through high-performance liquid chromatography fingerprinting. Figures 1A–D illustrates the reference compounds pachymic acid, cinnamaldehyde, 2-atractylenolide and glycyrrhizic acid. By comparing retention times with reference standards, the characteristic peaks corresponding to these compounds were identified in LGZG oral solution (Figure 1E).

### 3.2 Screening of optimal concentrations of the LGZG oral solution and OA + PA

**FIGURE 2:**
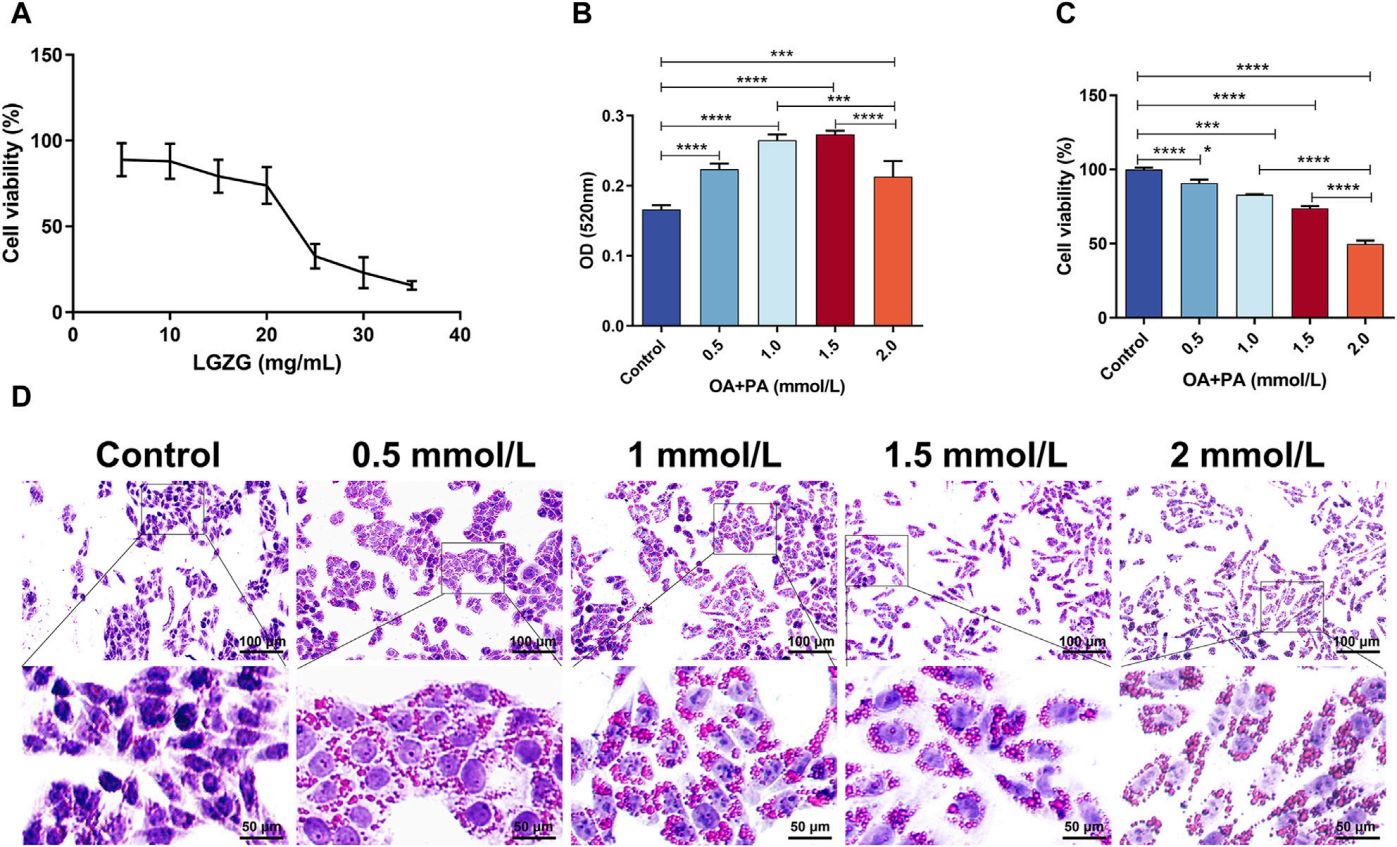
Screening of LGZG oral solution and OA + PA concentrations. **(A)** LGZG oral solution on LO2 cells activity assay. **(B)** OD of oil red staining of induced LO2 cells at 520 nm (n = 4). **(C)** Viability assay of oil red staining of induced LO2 cells (n = 5). **(D)** Oil red staining of LO2 cells induced by OA and PA. Data are presented as mean ± SD. ^**^ *p* < 0.01, ^***^ *p* < 0.001 and ^****^ *p* < 0.0001.

The effects of various concentrations of LGZG oral solution on the viability of LO2 cells were assessed to determine the effective drug intervention concentrations. The concentrations ranging from 0 mg/mL to 35 mg/mL were assessed. The Sulforhodamine B (SRB) method was used to determine the viability of LGZG-treated cells (Figure 2A). Following 24-h LGZG oral solution treatment, different levels of inhibition were observed in LO2 cells. To achieve reduced lipid deposition while maintaining high cell survival rates, the following concentrations were selected: low dose, 2.5 mg/mL; medium dose, 5 mg/mL; and high dose, 10 mg/mL. The concentration screening for inducing cell lipid deposition is shown in Figures 2B–D. OA and PA at concentrations of 1–2 mmol/L notably increased intracellular lipid deposition. However, 1.5–2 mmol/L concentrations markedly decreased the cell survival, indicating toxicity. Therefore, 1 mmol/L was selected as the inducing concentration for OA and PA, which is consistent with the findings of prior studies (Qiao et al., 2018).

### 3.3 LGZG oral solution inhibited lipid deposition and cytoprotection

**FIGURE 3:**
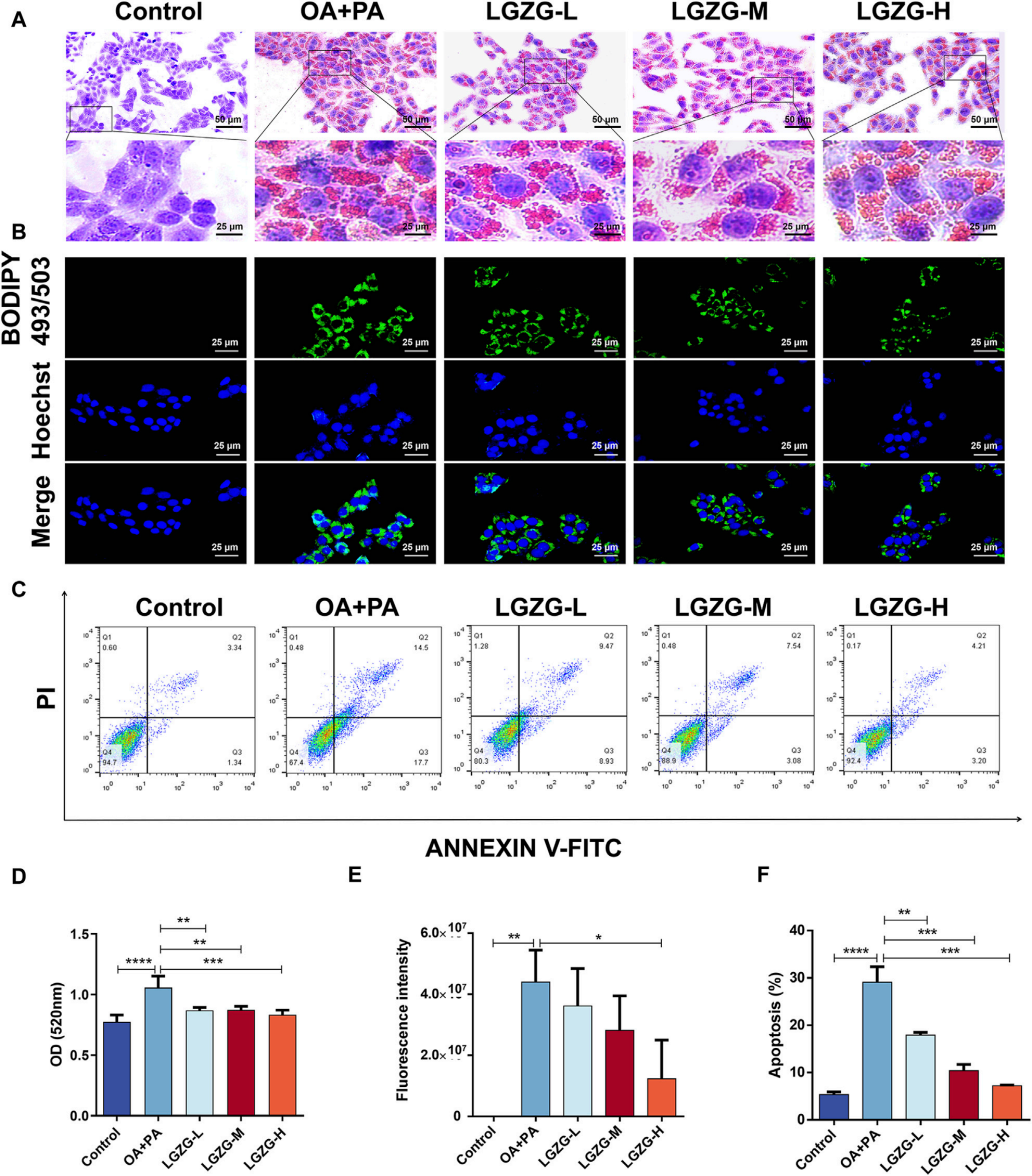
Effect of LGZG oral solution on lipid deposition and apoptosis in LO2 cells. **(A)** Oil red staining of lipid-deposited cells with LGZG oral solution. **(B)** BODIPY staining of lipid-deposited cells with LGZG oral solution. **(C)** Apoptosis of cells in different groups. **(D)** OD values of oil red staining (n = 4). **(E)** Quantitative analysis of fluorescence intensity (n = 3). **(F)** Different groups of total percentage of apoptotic cells (n = 3). Data are presented as mean ± SD. ^*^ *p* < 0.05, ^**^ *p* < 0.01, ^***^ *p* < 0.001 and ^****^ *p* < 0.0001.

The effect of LGZG oral solution on OA + PA-induced hepatocyte lipid accumulation was investigated by treating LO2 cells with 2.5, 5, and 10 mg/mL LGZG oral solution for 24 h. Intracellular lipid droplets were visualized using oil red O staining. Compared with the control group, lipid accumulation in OA + PA-induced hepatocytes markedly increased (Figures 3A, D). However, treatment with LGZG oral solution notably inhibited the aggregation of lipid droplets in LO2 cells. Additionally, BODIPY staining of lipid droplets in LO2 cells from different treatment groups revealed that the fluorescence intensity was highest in the OA + PA group, whereas the lowest intensity was in the LGZG-H group (Figures 3B, E). Flow cytometry analysis of the *in vitro* fatty liver model of LO2 cells showed an increased apoptosis rate in the OA + PA group; however, that in all LGZG-treated groups was reduced than that in the OA + PA group, with the LGZG-H group exhibiting the most notable decrease in the total apoptosis rate.

### 3.4 LGZG oral solution alleviated hepatocyte injury and oxidative stress

**FIGURE 4:**
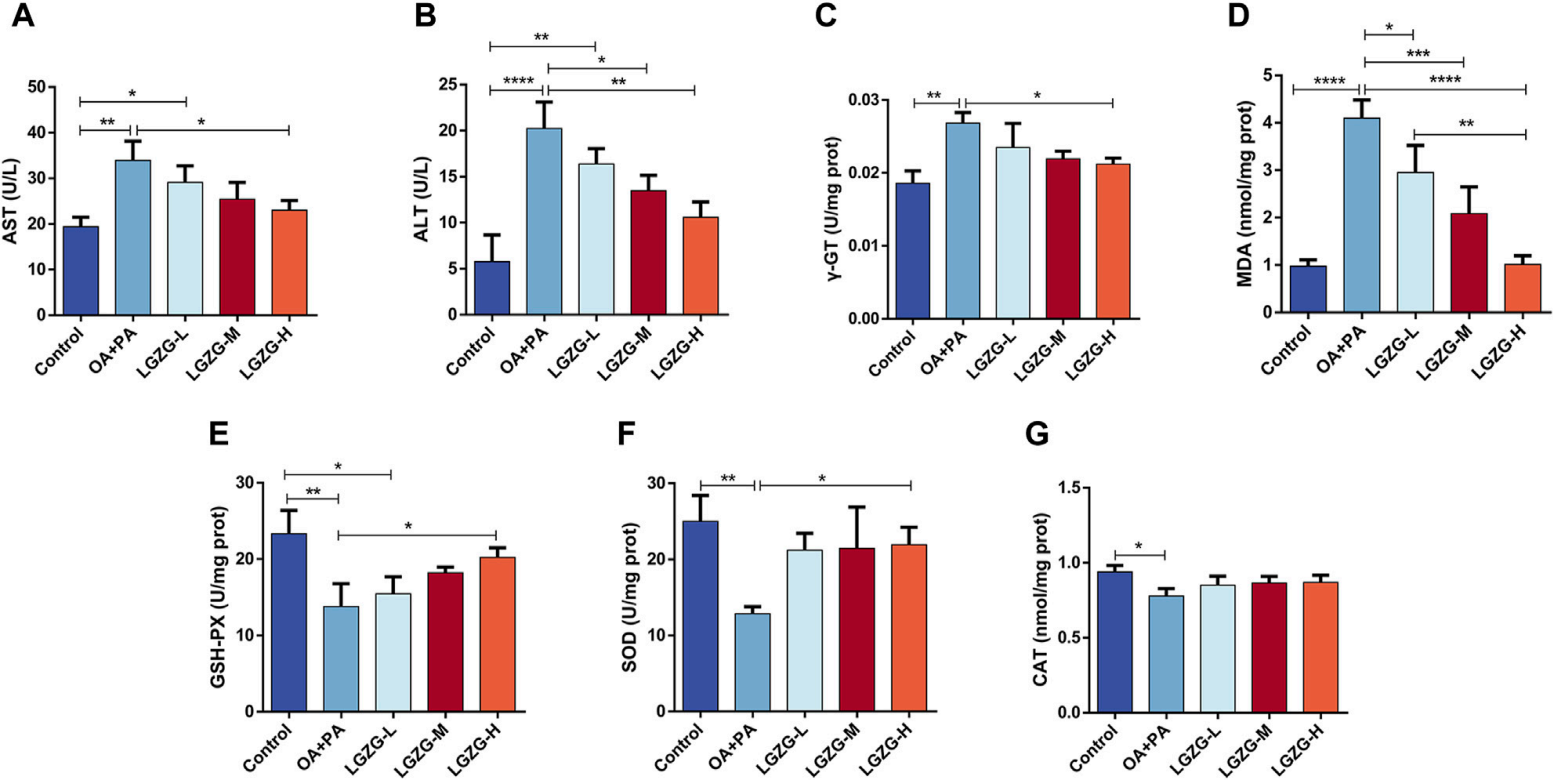
Effect of LGZG oral solution on some indices of fat depositing cells. **(A–G)** Measurement of AST, ALT, γ-GT, MDA, GSH-PX, SOD and CAT indicators. Data are presented as mean ± SD (n = 3). ^*^ *p* < 0.05, ^**^ *p* < 0.01, ^***^ *p* < 0.001 and ^****^ *p* < 0.0001.

In addition to the lipid accumulation, OA + PA increased the release of intracellular hepatic enzymes including alanine aminotransferase (ALT), aspartate aminotransferase (AST), and gamma-glutamyl transferase (γ-GT), which are markers of the liver cell damage (Fang et al., 2019; Liu et al., 2020). Notably, LGZG oral solution treatment reduced the enzyme levels. The effect of the LGZG oral solution on OA + PA-induced oxidative stress in LO2 cells was investigated by assessing various relevant indexes. The model group exhibited a significant increase in the malondialdehyde (MDA) content compared with that in the control group, indicating increased oxidative stress following the OA + PA treatment. LGZG-H oral solution treatment notably decreased the MDA content *versus* OA + PA (Figure 4D). Additionally, the expression of antioxidant enzymes glutathione peroxidase (GSH-PX), superoxide dismutase (SOD), and catalase was decreased in the model group compared with that in the control group, and LGZG oral solution treatment increased the levels of these enzymes (Figures 4E–G). These results confirmed that LGZG oral solution can effectively reduce lipid accumulation, mitigate hepatocyte injury, and alleviate oxidative stress in OA + PA-treated cells.

### 3.5 Protective effects of the LGZG oral solution in MASLD mice

**FIGURE 5:**
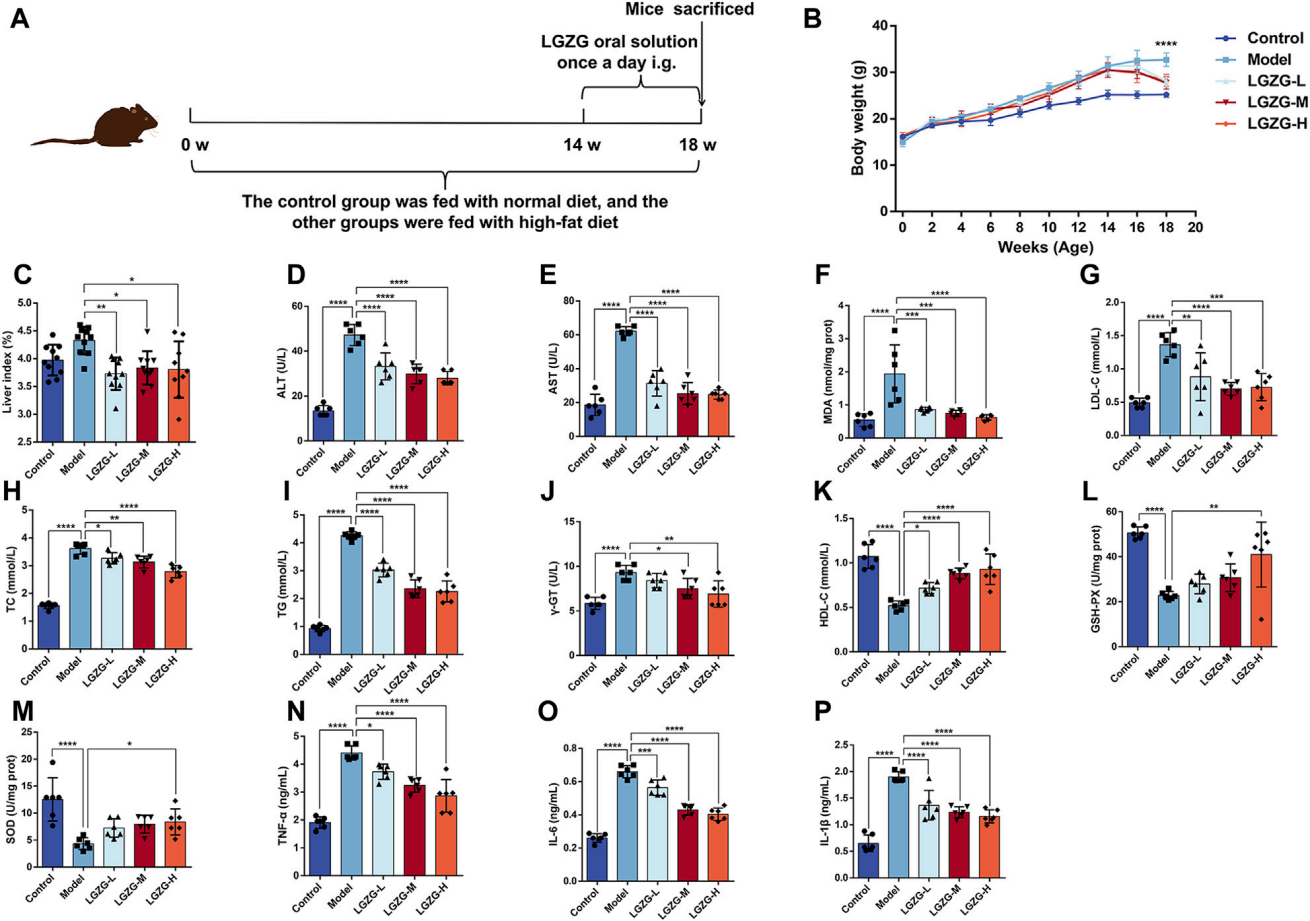
Effects of LGZG oral solution on MASLD mice. **(A)** Graphs depicting time of administration and time of tissue collection. **(B)** Changes in body weight of mice from 0 to 18 weeks (n = 10), ^****^ *p* < 0.0001, Control vs. Model and Model vs. LGZG-H. **(C)** The ratio of liver weight to body weight (n = 10). **(D–P)** Measurement of ALT, AST, MDA, LDL-C, TC, TG, γ-GT, HDL-C, GSH-PX, SOD, TNF-α, IL-6 and IL-1βindicators (n = 6). Data are presented as mean ± SD. ^*^ *p* < 0.05, ^**^ *p* < 0.01, ^***^ *p* < 0.001 and ^****^ *p* < 0.0001.

Herein, to investigate the effects of LGZG oral solution *in vivo*, the MASLD mouse model was established. Different concentrations of LGZG oral solution were administered starting from the 14th week for 4 weeks (Figure 5A). At the end of the experiment, changes in body weight and liver weight were measured for each group of mice. The model group exhibited a significant increase in body weight compared with that in the control group, and the LGZG-H group exhibited the most notable reduction in body weight and liver index compared with those in the model group (Figures 5B, C). The liver function, lipid metabolism, and oxidative stress markers post-treatment were assessed to investigate the effect of the LGZG oral solution on the progression of high-fat diet-induced MASLD in mice. ALT, AST, γ-GT, total cholesterol, triglyceride, low-density lipoprotein cholesterol, and MDA levels were considerably increased in the model group than those in the control group (Figures 5D–J), whereas high-density lipoprotein cholesterol, GSH-PX, and SOD levels were notably decreased in the model group (Figure 5K–M). LGZG oral solution treatment effectively reversed the high-fat diet-induced alterations in these marker levels. Because MASLD progression often involves an inflammatory response, interleukin (IL)-1β, IL-6, and tumor necrosis factor-α levels in the livers of mice from each group were assessed. The high-fat diet substantially increased the levels of these inflammatory markers, which were attenuated by LGZG oral solution treatment, particularly in the LGZG-H group (Figure 5N–P). These findings indicate that LGZG oral solution mitigates the development of high-fat diet-induced MASLD in mice.

### 3.6 LGZG oral solution alleviated hepatic steatosis in MASLD mice

**FIGURE 6:**
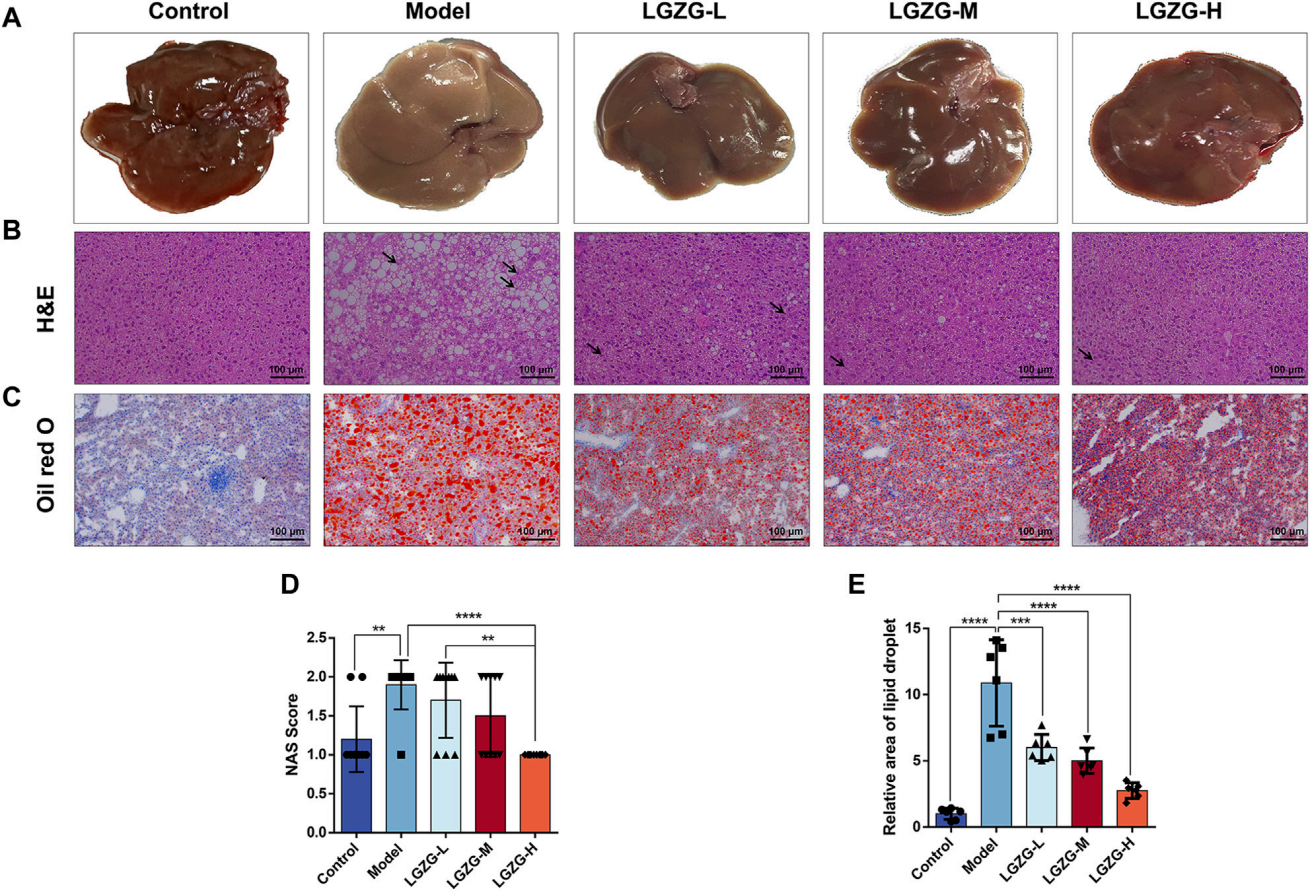
Effect of LGZG oral solution on liver histopathology in MASLD mice. **(A)** Representative images of livers (n = 10). **(B)** Representative images of liver tissue He staining (The black arrows indicate inflammatory infiltrating cells). **(C)** Representative images of liver tissue oil red O staining. **(D)** The NAS scores (n = 10). **(E)** Determination of the relative area of oil-red lipid droplets (n = 6). Data are presented as mean ± SD. ^**^ *p* < 0.01, ^***^ *p* < 0.001 and ^****^ *p* < 0.0001.

The livers of mice in the model group exhibited a yellowish and dull hue, whereas those in the LGZG-H group presented a ruddy and shiny appearance (Figure 6A). Histological analysis of the mouse liver tissues through the hematoxylin and eosin (HE) staining revealed the notably enlarged hepatocytes with visible lipid droplets in the model group compared with those in the control group (Figures 6B, D). Varying concentrations of LGZG oral solution reduced the hepatocyte swelling and fat droplets following administration. The arrows in Figure 6B indicate the pathological state of inflammatory cell infiltration, which was markedly reduced in the treatment group compared with that in the model group. Furthermore, the results of oil red O staining indicated a decrease in hepatic fat droplets in the LGZG groups compared with that in the model group (Figures 6C, E). Evaluation based on the NAS scoring criteria revealed that the NAS scores of all five groups of mice were below three points (Table 1), eliminating the possibility of nonalcoholic steatohepatitis. Notably, intervention with LGZG-H oral solution significantly decreased the NAS score of mice compared with that in the model group. Altogether, these *in vivo* findings indicate that LGZG oral solution exerts hypolipidemic, anti-inflammatory, and antioxidant effects in MASLD mice.

**TABLE 1:** The NAS scores of the five groups.

Group	Statistics of the number of mice in each group		
	1 score	2 score	3 score
Control	8	2	0
Model	1	9	0
LGZG-L	3	7	0
LGZG-M	5	5	0
LGZG-H	10	0	0

### 3.7 Metabonomic analysis of the LGZG oral solution in MASLD mice

**FIGURE 7:**
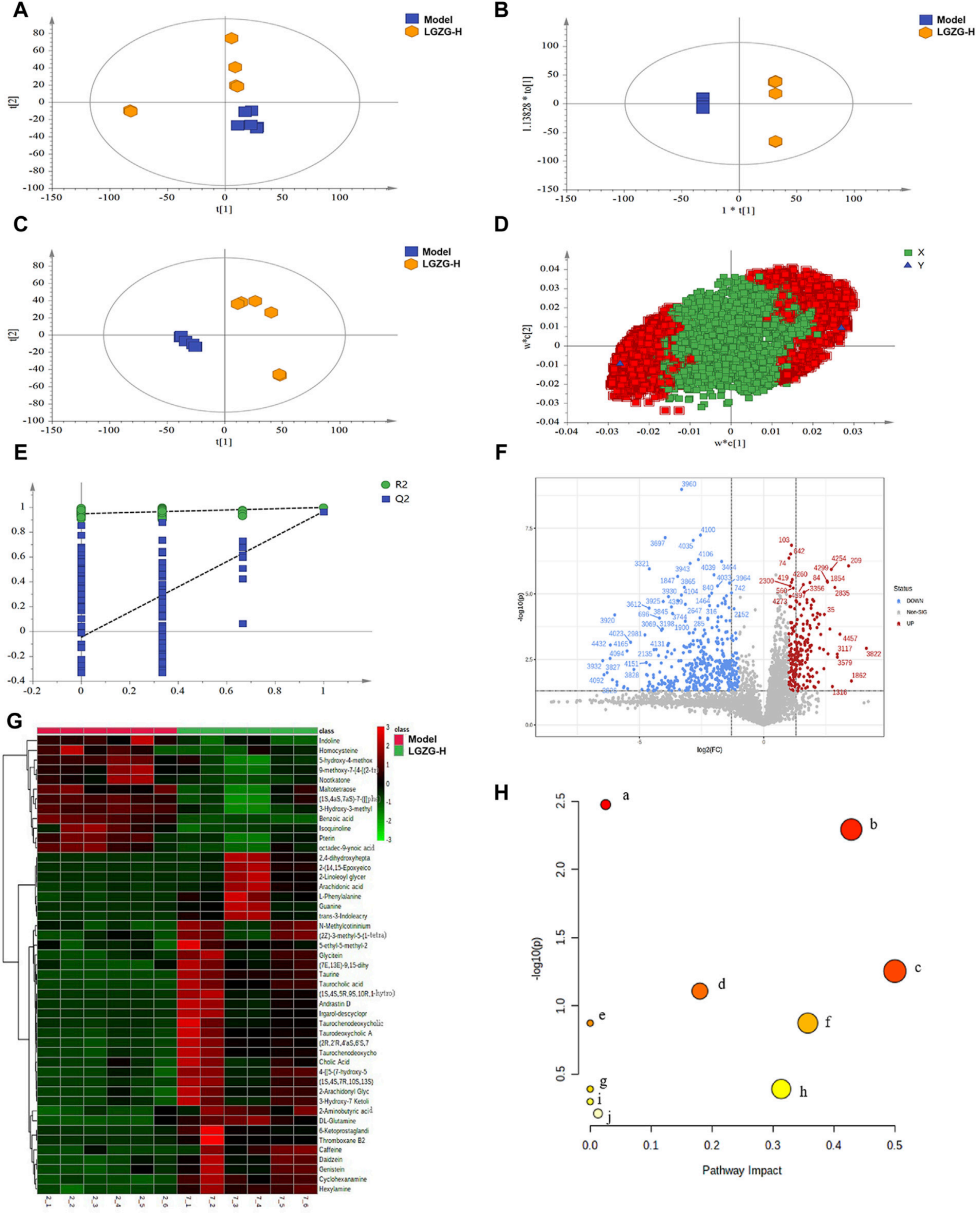
Effect of LGZG oral solution on serum metabolomics in MASLD mice. **(A)** Plot of PCA scores in the model and LGZG-H groups. **(B)** Plot of PLS-DA scores in the model and LGZG-H groups. **(C)** Plot of OPLS-DA scores in the model and LGZG-H groups. **(D)** PLS-DA model load diagram. **(E)** PLS-DA overfitting test. **(F)** Volcanograms of the model and LGZG-H groups. **(G)** Significantly different metabolite hierarchical clustering results between the model group and the LGZG-H group. **(H)** Metabolic pathway analysis of potential biomarkers. (a) Primary bile acid biosynthesis; (b) Taurine and hypotaurine metabolism; (c) Phenylalanine, tyrosine and tryptophan biosynthesis; (d) Cysteine and methionine metabolism; (e) Caffeine metabolism; (f) Phenylalanine metabolism; (g) Biosynthesis of unsaturated fatty acids; (h) Arachidonic acid metabolism; (i) Aminoacyl-tRNA biosynthesis; (j) Purine metabolism.

The efficacy of LGZG oral solution in the treatment of MASLD has been validated in previous studies in this paper. To investigate the mechanism of action of LGZG oral solution in inhibiting MASLD, a non-targeted metabolomics technology was used to analyze the metabolite levels in the mouse serum to identify differential metabolites. Principal component analysis (PCA), partial least squares (PLS)-discriminant analysis (DA), and orthogonal PLS-DA analyses were performed using the data from each group (Figures 7A–C). Notably, the separation between the model and LGZG-H groups was obvious in the positive ion mode. Figure 7D illustrates the model loading diagram of PLS-DA, identifying the differential metabolites. PLS-DA was performed with 200 random permutation tests, showing that R2 > Q2 and Q2 < 0, thereby indicating that the model was reliable and not overfitted (Figure 7E). The volcano plot revealed 47 initially identified differential metabolites in the positive ion mode, with 35 upregulated and 12 downregulated metabolites (Figure 7F). The hierarchical cluster analysis of the significant differential metabolites under the positive ion mode presented the distinct differences between the model and treatment groups, indicating variations in the types and quantities of differential metabolites between the groups (Figure 7G). Enrichment analysis was performed to identify the significant differential metabolites that were closely associated with the LGZG-H oral solution-mediated treatment of MASLD; the results are presented in bubble plots (Figure 7H). Compared with the model group, the LGZG-H treatment group exhibited considerable effects on metabolic pathways such as primary BA biosynthesis, taurine and hypotaurine metabolism, and phenylalanine, tyrosine, and tryptophan biosynthesis.

### 3.8 Effect of LGZG oral solution intervention on gut microbiota in MASLD mice

**FIGURE 8:**
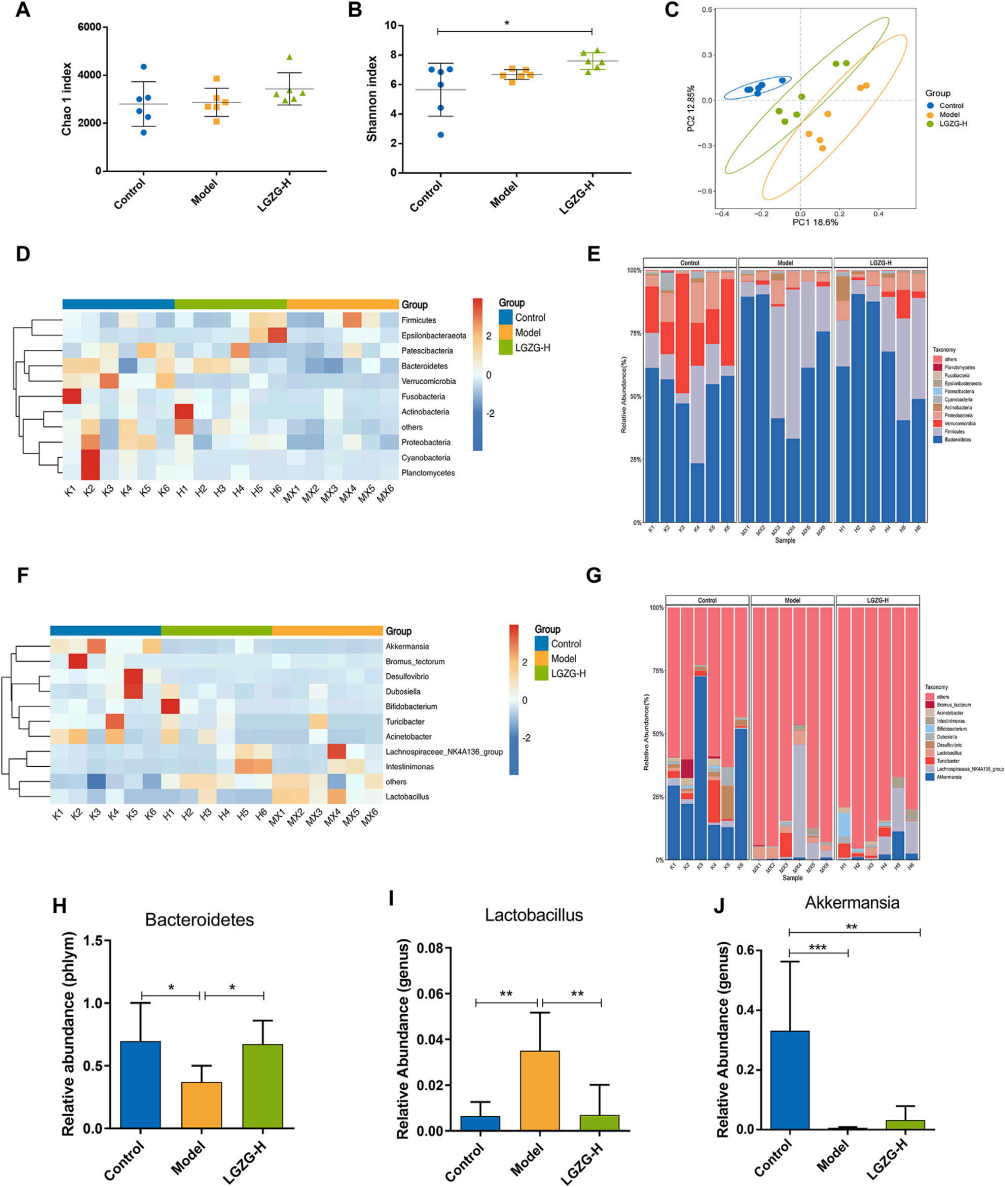
Effects of LGZG oral solution on the composition of gut microbiota. **(A)** Chao one index. **(B)** Shannon index. **(C)** The principal co-ordinates analysis (PCoA) plot. **(D, E)** Heatmaps and histograms of gut microbiota composition at the phylum level (top 10 relative abundance). **(F, G)** Heatmaps and histograms gut microbiota composition at the genus level (top 10 relative abundance). **(H–J)** Relative abundance of *Bacteroides*, *lactobacillus* and Akkermansia. Data are presented as mean ± SD (n = 6). ^*^ *p* < 0.05, ^**^ *p* < 0.01 and ^**^ *p* < 0.001.

Herein, the intricate relationship between microbial communities and BA metabolism, regarding the effects of LGZG oral solution on gut microbiota composition, was investigated. Alpha and beta diversity analyses of 16S rRNA gene sequencing data were performed to evaluate this relationship. The Chao1 index was employed to quantify the operational taxonomic units present in each sample, and the Shannon index provided insights into the species abundance and diversity of the gut microbiota. Notably, no considerable changes were observed in the Chao1 and Shannon indexes between the model and treatment groups (Figures 8A, B). However, beta-diversity analysis, including the principal coordinates analysis, revealed notable discrepancies in the gut microbiota composition among the three groups (Figure 8C). The relative abundances of the top 10 taxa at the phylum and genus levels in the three groups were compared to further explore the effects of LGZG oral solution on gut microbiota composition (Figures 8D–G). At the phylum level, *Bacteroidetes* exhibited a significantly higher abundance in the LGZG-H group compared with that in the model group (Figure 8H). At the genus level, the abundance of *Lactobacillus* was lower in the LGZG-H group than that in the model group (Figure 8I), whereas that of *Akkermansia* was higher in the LGZG-H group than that in the model group (Figure 8J).

### 3.9 Effect of LGZG oral solution intervention on BA metabolism in MASLD mice

**FIGURE 9:**
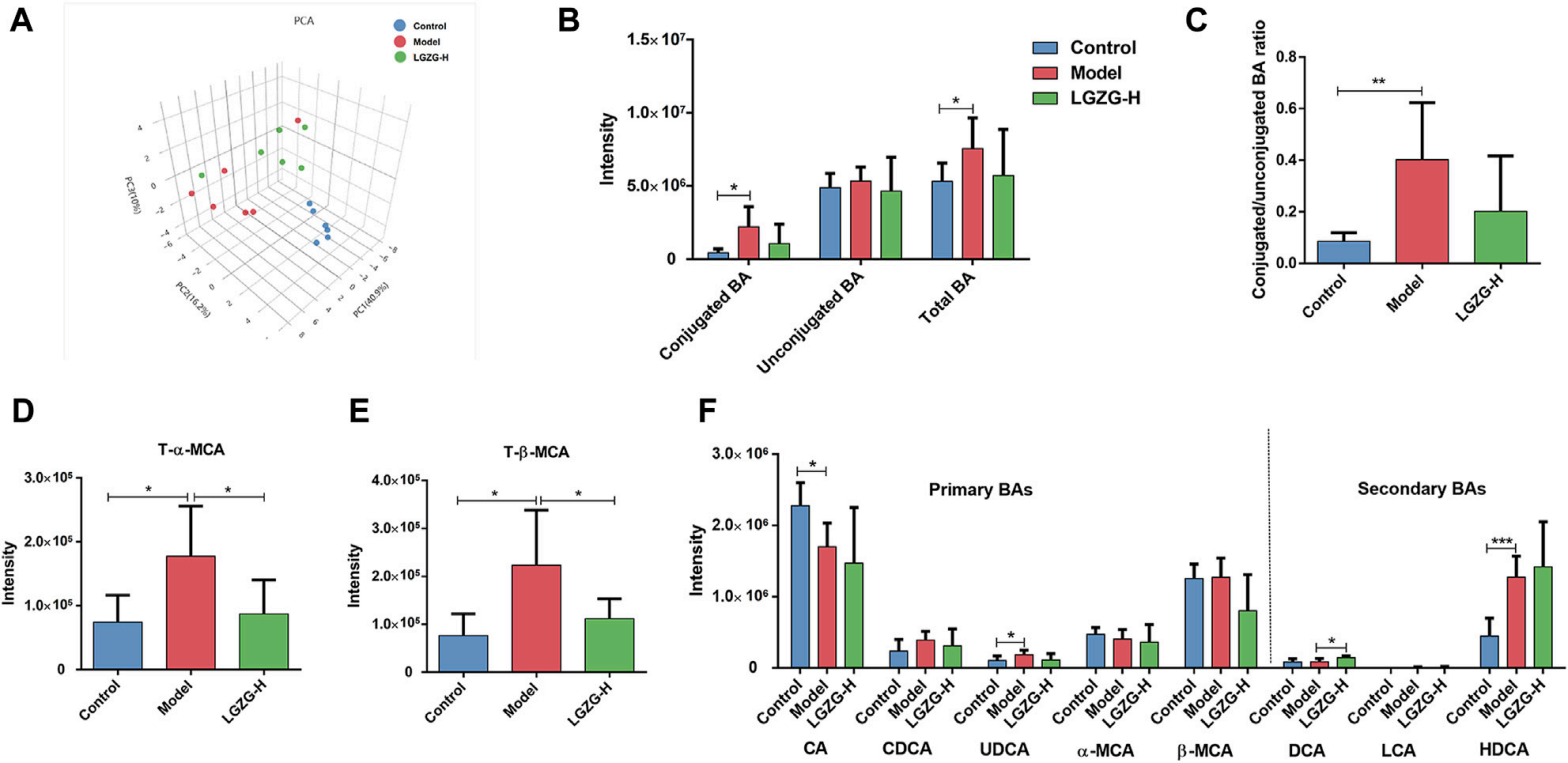
Effects of LGZG oral solution on the BA profiles. **(A)** Principal component analysis (PCA) plot. **(B)** The total BA level, conjugated BA level and unconjugated BA level. **(C)** The ratio of conjugated to unconjugated BAs. **(D)** The T-α-MCA level. **(E)** The T-β-MCA level. **(F)** The levels of typical primary and secondary BAs. Data are presented as mean ± SD (n = 6). ^*^ *p* < 0.05, ^**^ *p* < 0.01 and ^***^ *p* < 0.001.

Quantitative metabolomic analysis of the fecal BAs was performed to validate the discovered BA metabolic pathway. The PCA plots showed the different composition of BAs among the three groups (Figure 9A). The total and conjugated BAs showed a tendency to increase in the model group compared with that in the control group (Figure 9B). Additionally, the model group exhibited a significantly increased ratio of conjugated to unconjugated BAs (Figure 9C), whereas the LGZG-H group presented a lower conjugated/unconjugated BA ratio than that of the model group, but the difference was not significant. Interestingly, compared with the model group, fecal taurine-α-muricholic acid (T-α-MCA) and taurine-β-muricholic acid (T-β-MCA) were significantly decreased in the LGZG-H group (Figures 9D, E). The analysis of primary and secondary BAs showed that deoxycholic acid (DCA) levels were increased in the LGZG-H group compared with that in the model group (Figure 9F).

### 3.10 Effect of LGZG oral solution on the expression of BA metabolism-related proteins in MASLD mice

**FIGURE 10:**
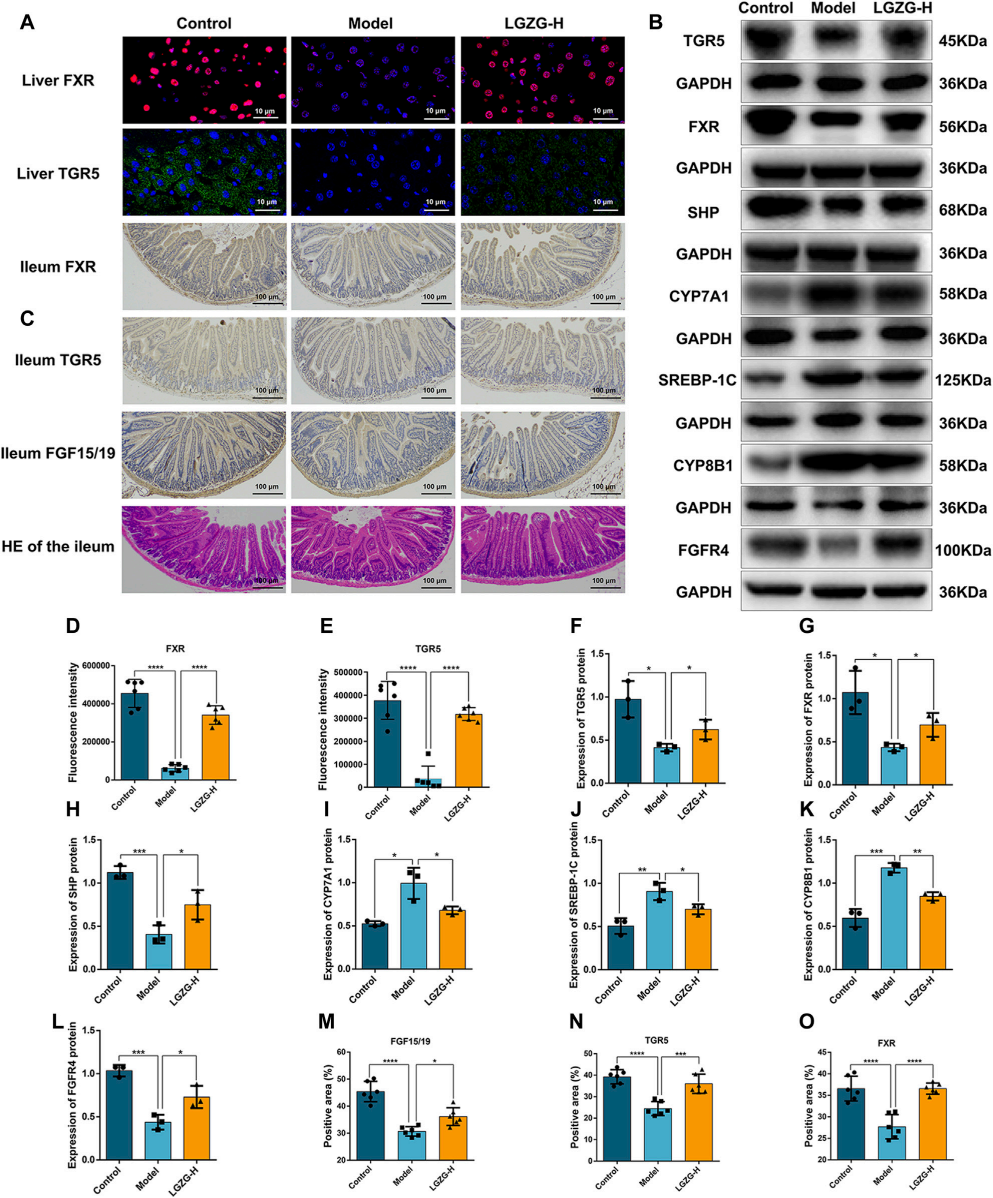
Effects of LGZG oral solution on hepatic lipid and bile acid synthesis. **(A)** The expression of FXR and TGR5 in the liver was estimated by immunofluorescence (n = 6). **(B)** The protein expression of FXR, TGR5, CYP7A1, SHP, CYP8B1, SREBP-1C and FGFR4 in the liver was determined by Western blotting (n = 3). **(C)** Immunohistochemical staining of FXR,TGR5, and FGF15/19 in the ileum and HE staining of ileum (n = 6). **(D, E)** Fluorescence intensity analysis of TGR5 and FXR (n = 6). **(F–L)** Grey-scale quantitative analysis of liver index. **(M–O)** Quantitative analysis of ileum index. Data are presented as mean ± SD. ^*^ *p* < 0.05, ^**^ *p* < 0.01, ^***^ *p* < 0.001 and ^****^ *p* < 0.0001.

Because LGZG oral solution showed considerable regulatory effect on BA metabolism, the expression of BA metabolism-related proteins in the liver and ileum was assessed through Western blotting, immunofluorescence, and immunohistochemistry. Immunofluorescence results showed that FXR and TGR5 expression was upregulated in the LGZG-H group compared with that in the model group (Figures 10A, D, E). The expression of BA metabolism-related proteins in the liver is shown in Figures 10B, F–L. Compared with the control group, the model group exhibited markedly downregulated expression of FXR, TGR5, SHP, and fibroblast growth factor receptor 4 (FGFR4) and notably upregulated expression of sterol regulatory element-binding protein-1c (SREBP-1c), CYP7A1, and cytochrome P450 family eight subfamily B member 1 (CYP8B1). However, the intervention of the LGZG-H oral solution reversed these changes. In the ileum, FXR, TGR5, and FGF15/19 were considerably downregulated in the model group, whereas their expression was upregulated in the LGZG-H group (Figures 10C, M, N). Additionally, HE results revealed that the structural disorganization and atrophy of the small intestinal epithelium in the model group of mice were improved following LGZG-H treatment, indicating the beneficial effect on intestinal epithelial villi damage (Figure 10C).

## 4 Discussion

LGZG decoction is a TCM composed of four botanical drugs. Herein, an LGZG oral solution was developed through water extraction and alcohol precipitation to enhance its stability and patient compliance while retaining the active compounds, without affecting its *in vivo* and *in vitro* anti-MASLD effects, including lipid deposition inhibition, anti-inflammatory effects, and antioxidant effects. In the LGZG oral solution, mannitol was used as a taste correction agent, and it did not affect the efficacy compared with that of LGZG decoction, indicating that the mannitol dose was sufficient to enhance the taste, without affecting MASLD. Non-targeted metabolomics analysis of the mouse serum revealed the potential effects of LGZG oral solution on BA metabolic pathways. Because gut microbiota plays an important role in regulating BA metabolism, the composition of gut microbiota was investigated. Notably, the LGZG oral solution could considerably increase the abundances of *Bacteroides* and *Akkermansia*, and reduce that of *Lactobacillus*. Reportedly, these bacteria are all involved in BA metabolism (Chen MJ. et al., 2021; Dong et al., 2021). Furthermore, the LGZG oral solution markedly reduced the conjugated/unconjugated BA ratio and taurine-α/β-muricholic acid (T-α/β-MCA) expression, increased DCA levels, and promoted FXR and TGR5 expression in the gut and liver of Ba-specific receptors, thereby upregulating SHP expression and downregulating CYP7A1 and SREBP-1c expression in the liver. Additionally, FGF15/19 expression in the gut was upregulated. These results suggest that the inhibition of hepatic lipid accumulation and BA stasis using the LGZG oral solution, which exerts therapeutic effects on MASLD, may be associated with the microbiota–BA–FXR/TGR5 axis.

The gut microbiota is composed of various bacteria, with Firmicutes, Bacteroidota, Proteobacteria, and Actinobacteria being the four main types. These microbial communities are crucially involved in metabolic processes within the body and considerably affect the onset and progression of diseases by regulating metabolites and metabolic pathways (Chen Y. et al., 2021). Prolonged consumption of a high-fat diet can alter the composition of gut microbiota (Zhang and Yang, 2016), notably affecting the Firmicutes/Bacteroidota (F/B) abundance ratio. A decrease in the F/B ratio results in an increase in beneficial bacteria and a decrease in pathogenic bacteria, ultimately improving the integrity of the intestinal barrier (Feng et al., 2022). The gut microbiota mainly produces BA, short-chain fatty acids, and other metabolites (Guzior and Quinn, 2021; Nogal et al., 2021). In the small intestine, microbes transform BAs through various processes, including BSH-mediated hydrolysis of bound BAs to free BAs and glycine or taurine and 7α/β-dehydroxylation-mediated conversion of primary BA to secondary BA (Ridlon et al., 2016). BSH-producing microbes, such as *Bacteroides* and *Lactobacillus*, are involved in transforming BAs (Chen MJ. et al., 2021). The abundance of *Lactobacillus* has been reported to be considerably increased, whereas that of Bacteroidota to be markedly decreased in the fecal samples of mice fed with a high-fat diet (Sun et al., 2022). Herein, LGZG oral solution intervention decreased the abundance of *Lactobacillus* and increased the abundance of Bacteroidota in mice. Additionally, although studies on the role of *Akkermansia* in BA metabolism processes are limited, this genus has been associated with increased levels of unconjugated BAs in the gastrointestinal tract (Dong et al., 2021).

BAs are derived from the catabolism of cholesterol and are the main metabolites of the bile. They enter the intestine through the bile ducts and participate in the enterohepatic cycle. A classical synthesis pathway of BA can produce cholic acid (CA) and chenodeoxycholic acid (CDCA). The classical synthetic pathway is catalyzed by CYP7A1, which generates CA under the action of CYP8B1 and CYP27A1, and CDCA in the absence of CYP8B1 (Thomas et al., 2008). CA and CDCA are primary BAs, which combine with glycine and taurine to form bound BAs; most of the bound BAs are reabsorbed at the end of the ileum, while the rest are excreted (Yang et al., 2021). Hepatocyte injury leads to abnormal BA metabolism in the body, resulting in increased levels of total BA. Different BAs exhibit different affinity and transcriptional activation of FXR, with CDCA, DCA, and lithocholic acid being the potent natural ligands for FXR, and the conjugated BAs, such as T-α-MCA and T-β-MCA, being the natural antagonists of FXR that promote BA synthesis and enterohepatic circulation (Panzitt and Wagner, 2021). The results of this study showed that LGZG oral solution considerably reversed the high-fat diet-induced alterations in the intestinal flora and altered BA profiles, including a notable increase in DCA levels, an FXR and TGR5 activator, and a marked decrease in T-α/β-MCA levels, an FXR inhibitor. Altogether, these results suggest that LGZG oral solution promotes BA metabolism. Additionally, BA acts on FXR and TGR5 to regulate the activity of CYP7A1, reducing BA synthesis and BA-mediated injury in the liver. FXR inhibits the activity of CYP7A1 and CYP8B1 by inducing SHP expression in the liver. FXR activation considerably reduced TG and cholesterol levels in the liver of the model group, inhibiting the expression of fat-synthesizing genes, and thus, inhibiting SREBP1 expression. In contrast, FXR receptors in the intestinal tract were upregulated, inducing the expression of the target gene FGF15/19. FGF15/19 enters the liver *via* the portal vein and acts through the hepatocyte membrane receptor FGFR4, activating a series of signaling molecules, and ultimately inhibiting hepatic CYP7A1 expression (Chiang and Ferrell, 2020). TGR5 activation exhibits hepatoprotective effects in the presence of liver injury and BA deposition. Reportedly, treating high-fat diet-fed mice with INT-777, a TGR5-specific agonist, reduced body weight gain and serum TG levels (Keitel et al., 2019b). Studies have shown that activating BA receptors FXR and TGR5 pathway could play a role in regulating glucose and lipid metabolism, inflammatory response, cell proliferation, and apoptosis (Stepanov et al., 2013). This study showed that the LGZG oral solution effectively increased the FXR and TGR5 expression in the liver and intestinal tissues of MASLD mice. Additionally, following the LGZG treatment, the expression of SHP, FGFR4, and FGF15/19 were upregulated, whereas that of CYP7A1, CYP8B1, and SREBP-1C was downregulated. Altogether, these findings indicate that LGZG oral solution could potentially modulate the progression of MASLD *via* the FXR/TGR5 pathway.

## 5 Conclusion

In summary, the LGZG oral solution is a promising therapeutic approach to regulating lipid metabolism, inflammation, and oxidative stress to reduce pathological damage in patients with MASLD. Its potential mechanism of action may involve modulating gut microbiota and BA metabolism, thereby affecting the FXR/TGR5 pathway. However, additional research is required to fully elucidate the precise molecular mechanism and target of this therapeutic effect.

## Data Availability

The data presented in the study are deposited in the NCBI repository, accession number PRJNA1145154.

## Abbreviations

LGZG-H, high dose Lingguizhugan oral solution; LGZG-M, middle dose Lingguizhugan oral solution; LGZG-L, low dose Lingguizhugan oral solution; CA, cholic acid; CDCA, chenodeoxycholic acid; DCA, deoxycholic acid; T-α/β-MCA, taurine-α/β-muricholic acid; FGF15/19, fibroblast growth factor 15/19; FXR, farnesoid X receptor; CYP7A1, cholesterol 7α-hydroxylase; TGR5, takeda G protein-coupled receptor 5; SHP, short heterodimer partner; SREBP-1c, sterol regulatory element-binding protein-1c; CYP8B1, cytochrome P450 family 8 subfamily B member 1; FGFR4, fibroblast growth factor receptor 4; HFD, high-fat diet.
